# Diagnosis and early detection of CNS-SLE in MRL/lpr mice using peptide microarrays

**DOI:** 10.1186/1471-2172-15-23

**Published:** 2014-06-07

**Authors:** Stephanie Williams, Phillip Stafford, Steven A Hoffman

**Affiliations:** 1 Neuroimmunology Labs, School of Life Sciences, Arizona State University, Tempe, AZ 85287-4501, USA; 2 Center for Innovations in Medicine, BioDesign Institute, School of Life Sciences, Arizona State University, Tempe, AZ, USA

**Keywords:** Lupus, CNS-lupus, Microarray, Diagnostic, Predictive, Brain-reactive autoantibodies

## Abstract

**Background:**

An accurate method that can diagnose and predict lupus and its neuropsychiatric manifestations is essential since currently there are no reliable methods. Autoantibodies to a varied panel of antigens in the body are characteristic of lupus. In this study we investigated whether serum autoantibody binding patterns on random-sequence peptide microarrays (immunosignaturing) can be used for diagnosing and predicting the onset of lupus and its central nervous system (CNS) manifestations. We also tested the techniques for identifying potentially pathogenic autoantibodies in CNS-Lupus. We used the well-characterized MRL/lpr lupus animal model in two studies as a first step to develop and evaluate future studies in humans.

**Results:**

In study one we identified possible diagnostic peptides for both lupus and altered behavior in the forced swim test. When comparing the results of study one to that of study two (carried out in a similar manner), we further identified potential peptides that may be diagnostic and predictive of both lupus and altered behavior in the forced swim test. We also characterized five potentially pathogenic brain-reactive autoantibodies, as well as suggested possible brain targets.

**Conclusions:**

These results indicate that immunosignaturing could predict and diagnose lupus and its CNS manifestations. It can also be used to characterize pathogenic autoantibodies, which may help to better understand the underlying mechanisms of CNS-Lupus.

## Background

Systemic lupus erythematosus (SLE) is an autoimmune disease that affects many organs including the joints, kidneys and brain [1]. Some of the symptoms include arthritis, rashes, seizures, and psychoses. One of the characteristics of lupus is the detection of autoantibodies to numerous different antigens in the body [2,3]. The brain is one of the affected organs, causing neuropsychiatric manifestations in 31% to 70% of lupus patients, resulting in cognitive impairment and psychoses [4-7]. We have hypothesized that there are brain reactive autoantibodies that bind to integral membrane proteins of the brain and this interaction is partly responsible for some of the neuropsychiatric manifestations seen in lupus [8,9]. These BRAA can enter the brain through increased permeability of the blood–brain barrier as lupus progresses or are produced in the brain once antibody producing cells enter the brain [10].

Our model of lupus is the MRL/lpr mice (Jackson Lab, Bar Harbor, ME). These mice develop lupus symptoms after 2 months of age and have 50% mortality at about 5–6 months of age. The MRL/lpr have manifestations similar to humans including rashes, swollen joints and neurobehavioral manifestations [11,12]. A mutant of the *fas* gene, the *lpr* gene, is thought to help accelerate lupus-like symptoms in these mice. Because of the similarity to human lupus, the MRL/lpr mouse is an excellent model of SLE and has been used by many other researchers as their model of choice [11,12].

The manifestations of lupus resemble the manifestations of other diseases, making accurate diagnosis difficult. Physicians use a set of 11 different criteria and patients must satisfy 4 out of 11 to be diagnosed as having lupus [13]. Antinuclear antibodies and anti-DNA autoantibodies have been used as some of the markers for the diagnosis of lupus [14]. However, these markers are not specific for lupus. Therefore, being able to correctly diagnose and even predict the onset of lupus and its CNS manifestations is of high importance due to the current inability to do so [14].

We have multiple goals in this report. The first goal is to diagnose lupus, and CNS lupus, using sera, in a reliable and rapid manner. We tested the idea that we could do this using immunosignaturing [15]. There is mounting evidence that this technique may be useful to diagnose other CNS diseases such as Alzheimer’s [16,17]. Our second goal was to predict lupus onset, and specific CNS manifestations, pre-symptomatically. There are low concentrations of autoantibodies present in the sera even before clinical signs of lupus. If some autoantibodies predict the onset of lupus, and specific CNS manifestations, detection using immunosignaturing is possible. Identification of potential predictive peptides for specific CNS manifestations would be unique to this study. We and others have used the forced swim test as a measure of depressive like behavior in the MRL/lpr model [1,2,18]. In the current study we utilized this test to indicate CNS dysfunction, however, it should be noted that this test is only one measure and therefore does not represent all CNS dysfunction. It is expected that other peptide subsets generated using our microarray techniques will probably correlate with other measures of CNS dysfunction.

Our third goal was to obtain preliminary information on the utility of this technique for future studies in characterizing the antigenic targets of potentially pathogenic brain-reactive autoantibodies. The random-sequence peptide microarray was used to identify peptides reactive to antibodies against BRAA. Peptide sequences were analyzed using the Guitope computer program [19] to identify potential protein targets. As an initial test, we created five monoclonal BRAA from an MRL/lpr mouse with behavioral dysfunction to identify likely targets of these monoclonal BRAA.

The latter is important because determining the identity of BRAA targets will allow us to better understand their potential functional effects, and whether they may be mediating neuropsychiatric manifestations. These might also provide new therapeutic targets. For example, one group of researchers has found an autoantibody that reacts with double-stranded DNA and the NMDA receptor [20]. This autoantibody resulted in cognitive deficits in their murine model, suggesting that this NMDA receptor autoantibody could be responsible for CNS manifestations [21]. Another researcher found an autoantibody that is cross-reactive with the dynamin-1 protein that also altered the behavioral performance of their autoimmune murine model in comparison to controls [22]. These research findings are, however, relatively random. Our techniques should provide a reliable method for identifying such potentially pathogenic BRAA and their targets.

Overall, our microarray technology should be more accurate in diagnosing and predicting lupus and its CNS manifestations, and allow for the optimum identification of potentially pathogenic BRAA and their target antigens. We were using a well validated animal model in this study in order to see if future studies with humans are warranted. We believe that our findings warrant human studies.

## Methods

### Animals

We used female MRL/lpr, MRL/mpJ (develops lupus-like disease at about 12 months of age) and C3H/HeJ mice. Females were employed instead of males since in humans there is a sex-related difference where onset is more predominant in females, however for the MRL/lpr mice no overt sex-related differences exist, although there is some suggestion that a propensity does exist at the genetic level [23]. In Study 1, we used 3–6 MRL/lpr, MRL/mp and C3H/HeJ at 4 months and in Study 2 we increased the number to 9–10 MRL/lpr and MRL/mp at 1.5 and 4 months. Blood was collected at 1.5 and 4 months in Study 2, but only at 4 months in Study 1. The mice were obtained from Jackson Laboratory (Bar Harbor, ME) and individually housed under standard conditions for both studies. The light cycle was from 6:00 A.M. to 6:00 P.M. They were given food and water ad libitum. In both studies the behavioral tests were performed similarly at 8:00 P.M.

The mice were sacrificed with an intraperitoneal (IP) injection of Nembutal sodium solution. The blood was collected via cardiac exsanguination and allowed to coagulate in microcentrifuge tubes. The tubes were centrifuged for 10 minutes at 8500 rpm (5200 × g). The serum was aliquoted and frozen at −50°C. The spleen weight was divided by the body weight of that mouse to control for differences in body weight.

Animals were maintained in university facilities fully accredited by AAALAC and are registered with the USDA APHIS (Registration # 86-R-0002). An assurance is on file with the Office for Laboratory Animal Welfare (#3217-01). Animal husbandry programs and protocol review are in compliance with NIH and USDA standards. The protocols used in the animal studies were approved by the Institutional Animal Care and Use Committee at Arizona State University.

### Behavioral testing

Our battery of behavioral tests included the forced swim test and the sucrose preference test. The forced swim test is used to analyze anti-depressants by measuring float time and the sucrose preference test can assess anhedonia (i.e., the desire to seek out pleasurable stimuli) through quantifying consumption. Both tests have been used with the MRL/lpr mice to indicate emotional dysfunction [1]. The sucrose preference test has been described previously [2], except the mice were given 7 ml of the 4% sucrose solution for 1 hour each day for three days in the testing phase. The forced swim test has also been previously described [2]. The MRL/lpr mice are expected to have high float times and low sucrose consumption. In this manuscript, the results of the sucrose preference test were determined to be inconclusive and were not reported.

### Immunological assessment

#### Integral membrane protein preparation and ELISA for anti-DNA and BRAA

The technique to extract integral membrane proteins from the brain has been previously described [24]. A two month C3H/HeJ brain was used in the BRAA ELISA. Once the integral membrane proteins were extracted they were suspended in phosphate buffered saline (PBS) and protein concentration determined using the BCA Assay Kit (Pierce, USA). Anti-DNA and brain-reactive autoantibody levels were determined using previously described ELISA protocols [2,25,26]. The optical density of the control wells (the even wells) were subtracted from the optical density of the odd wells to give an S-value that showed the levels of anti-DNA antibodies (or BRAA). Some S-values may be negative, since the S-values are a relative measure.

### Microarray analysis

Microarrays containing 10,000 random-sequence 20-mer peptides were obtained from the Center for Innovations in Medicine, Biodesign Institute, Arizona State University [15]. Manufacture of the arrays were described previously [27]. Microarrays were processed in a Tecan HS 4800 Pro (Tecan, Mannedorf, Switzerland). The hybridization station performed these steps: arrays were pre-washed in PBS buffer and exposed to a 1:500 dilution of serum or a 10nM purified monoclonal antibody solution and detected with 5nM biotinylated anti-idiotype secondary antibody, which was then exposed to 5nM streptavidin conjugated to Alexafluor 647 fluorophore (Alexafluor 555 fluorophore for mice sera). Arrays were washed to remove any unbound material and scanned in a Perkin Elmer ScanArray laser scanner at 650 nm and 10um resolution. Tiff images were aligned to the corresponding .gal file and a .gpr file was produced containing the intensity values corresponding to the appropriate peptide. Typical technical reproducibility was >0.80 Pearson’s Correlation Coefficient for all 10,000 peptides.

Each peptide on the microarray was synthesized and quality tested by Alta Biosciences (Birmingham, UK). The sequence, mass spectrometry of each peptide, and other chemical characteristics are known. No peptide with <80% purity by mass spectrometry was used in these arrays. Our microarray data have been deposited in GEO under accession numbers GSE57388 (http://www.ncbi.nlm.nih.gov/geo/query/acc.cgi?acc=GSE57388) and GSE57389 (http://www.ncbi.nlm.nih.gov/geo/query/acc.cgi?acc=GSE57389).

### Monoclonal antibody production

Monoclonal BRAA were produced using a ClonaCell®-HY Hybridoma Kit purchased from STEMCELL Technologies (Vancouver, Canada). The protocol used has been described in the manual. Mouse myeloma cells (Sp2/0-Ag14 – ATCC (USA)) were fused with spleen cells from MRL/lpr #2. Cell viability was determined using trypan blue. Visible colonies on the petri dishes were plated in 96-well plates and tested for the presence of BRAA using ELISAs and if positive were transferred to 24-well plates. If the cells were still positive, they were cultured in petri dishes and then stored at −80°C.

### Western blotting

The Western blotting protocol has been described previously [2]. The blots were cut into strips and incubated with different primary antibodies. For some of the strips more wash steps were necessary to better see the bands. The size of the bands was calculated using a linear plot of the molecular weights of the protein marker and distance traveled (Fermentas, USA).

### Immunohistochemistry

Mid-brain sections from a 4 month C3H/HeJ control mouse were used to determine the binding location of the 5 different monoclonal BRAA as well as sera from LPR #2 (the mouse used to create the monoclonals). The immunohistochemistry technique used was previously described [2]. The slides were observed using a Leitz Laborlux 12 microscope and pictures were taken using a Nikon camera at 4× and 250× magnification.

### Statistics

Analytical methods for conventional expression microarrays were used for the immunosignature microarrays and no unusual biases were noted. The arrays yielded 14% average slide-to-slide Coefficient of Variance, and a 1.3-fold minimum detectable fold-change at the 95^th^ percentile, near the reproducibility of typical commercial microarrays. All experiments were done with triplicate technical and triplicate biological replicates (9 samples per condition). Tests were done on pooled sera from replicate mice but each mouse was also analyzed independently to determine mouse-to-mouse variance as well as the robustness and representative nature of each immunosignature. Numerical data from the gpr files were exported to GeneSpring 7.3.1 (Agilent, Palo Alto, CA) or R (CRAN GNU open-source) for analysis. Slides were median normalized and log-10 transformed for analysis. Statistics typically used standard t-test between case and control using the Benjamini and Hochberg False Discovery Correction Rate set at 5%. Technical replicates were averaged prior to conducting the t-test.

**Figure 1:**
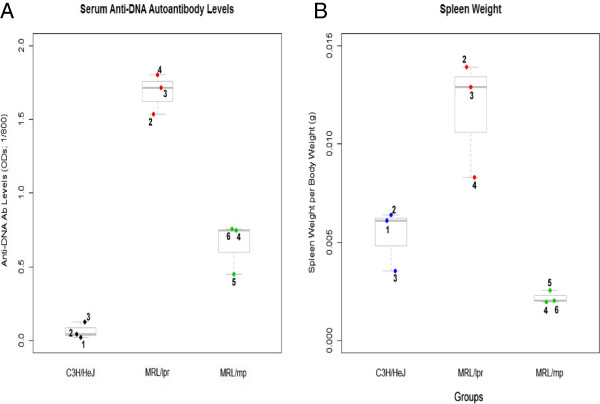
**Immunological assessment for Study 1. (A)** Three randomly selected mice from each of the groups were used for immunologic assessments (the numbers next to each dot represent the mouse number). ANOVA showed that anti-DNA autoantibody levels was significantly different between the groups (F = 112.953, p < 0.001) and using post-hoc analysis at p < 0.001 the 4 month MRL/lpr had significantly greater anti-DNA antibody levels compared to controls (measured at 1:800 serum dilution). **(B)** For the spleen weights there was a significant difference between the groups (F = 18.365, p < 0.003) and post-hoc analysis at p < 0.007 revealed that the 4 month MRL/lpr had significantly greater spleen weights (per body weight) compared to the MRL/mp and C3H/HeJ. In the box plot the middle line is the 50^th^ percentile, the top of the box is the 75^th^ percentile and the bottom of the box is the 25^th^ percentile.

**Figure 2:**
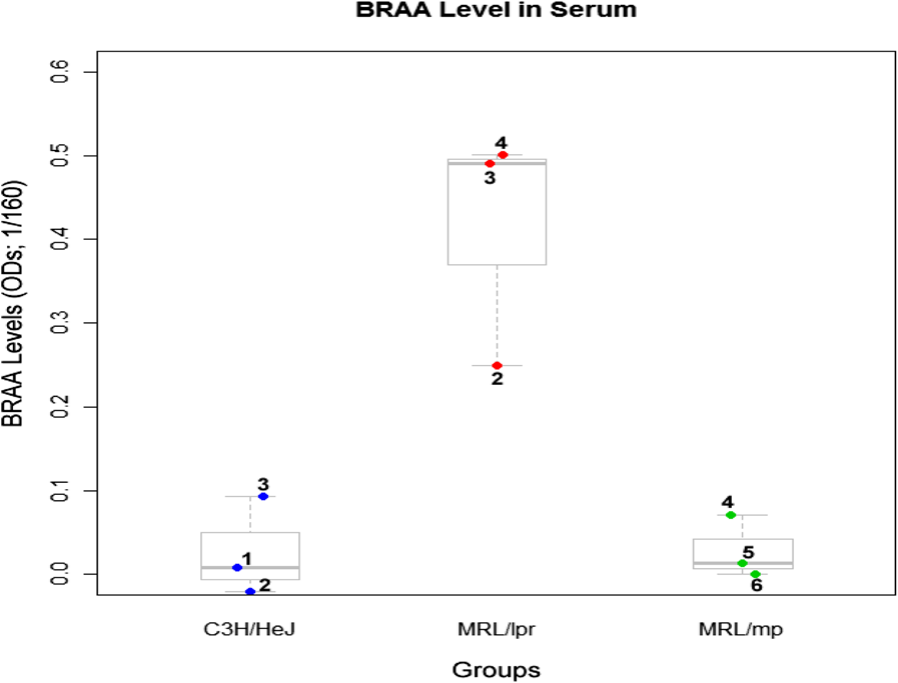
**Serum BRAA Levels for Study 1.** ANOVA of the BRAA levels revealed a significant difference between the groups (F = 14, p < 0.005). Post-hoc analysis at p < 0.004 showed that the 4 month MRL/lpr had significantly greater BRAA levels in comparison to the MRL/mp and C3H/HeJ.

**Figure 3:**
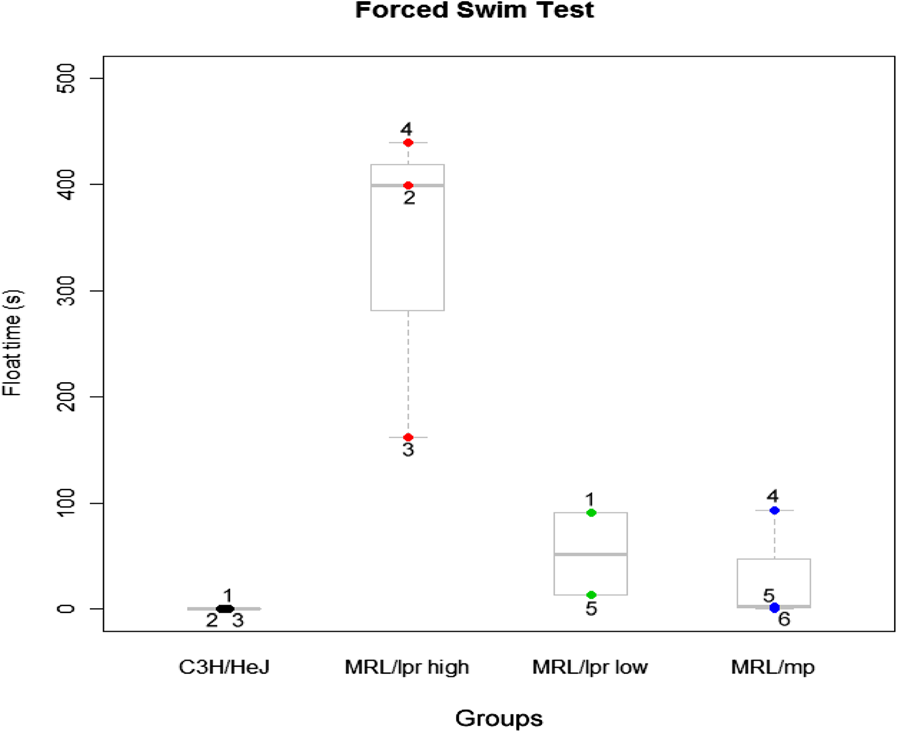
**Forced Swim Test Study 1 – Group Separation within the MRL/lpr by Neurobehavioral Manifestations.** The five MRL/lpr mice were split into two groups with mice 2, 3 and 4 grouped as high floaters and 1 and 5 were low floaters. There was an overall significant difference between the groups (F = 9.2, p < 0.008). Post-hoc analysis at p < 0.010 revealed that the MRL/lpr high floaters were significantly different from the MRL/lpr low floaters, the MRL/mp and C3H/HeJ. The MRL/lpr low floaters were not significantly different from the MRL/mp and C3H/HeJ.

**Figure 4:**
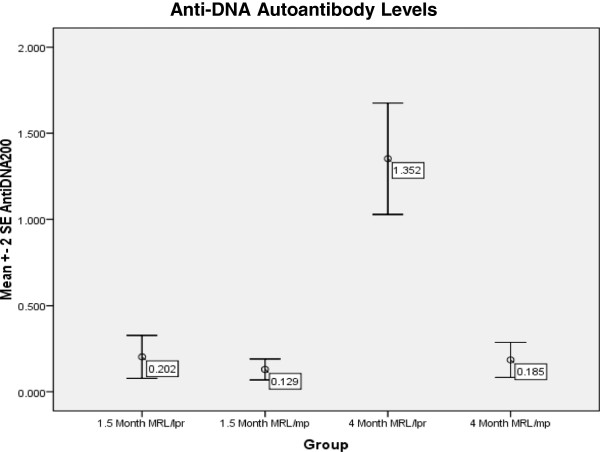
**Immunological Assessment for Study 2 – Anti-DNA Autoantibody Levels.** Anti-DNA autoantibody levels was measured for the MRL/lpr (N = 9-10) and MRL/mp at 1.5 and 4 months of age. ANOVA revealed that there was a significant difference between the groups (F = 44.067, p < 0.001) and LSD post-hoc analysis at p < 0.001 showed that the 4 M MRL/lpr had significantly greater anti-DNA autoantibody levels compared to the 4 M MRL/mp, 1.5 M MRL/lpr and 1.5 M MRL/mp.

**Figure 5:**
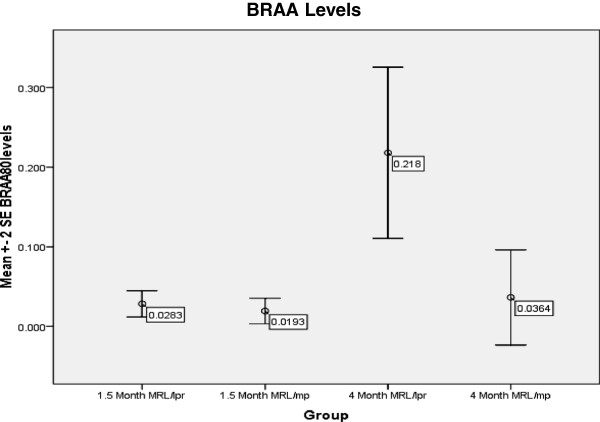
**Immunological Assessment for Study 2 – BRAA Levels.** BRAA levels was measured and ANOVA revealed that there was a significant difference between the groups (F = 9.746, p < 0.001). Utilizing post-hoc analysis at p < 0.001 the 4 M MRL/lpr had significantly greater BRAA levels compared to the 4 M MRL/mp, 1.5 M MRL/lpr and 1.5 M MRL/mp.

**Figure 6:**
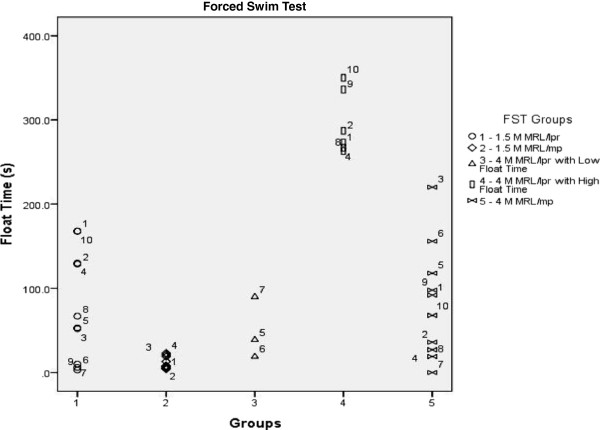
**Forced Swim Test Study 2 – Group Separation within MRL/lpr by Neurobehavioral Manifestations.** The forced swim test data was re-plotted to separate the 4 M MRL/lpr into two groups (3 and 4) based on their float times. The groups were as follows: 1 – 1.5 M MRL/lpr, 2 – 1.5 M MRL/mp, 3 – 4 M MRL/lpr low floaters, 4 – 4 M MRL/lpr high floaters and 5 – 4 M MRL/mp. Group 3 included mice 5, 6 and 7 and group 4 included mice 1, 2, 4, 8, 9 and 10. ANOVA revealed that there was a significant difference between the groups (F = 30.253, p < 0.001) and post-hoc analysis at p < 0.001 showed that the 4 M MRL/lpr low floaters were only significantly different from the 4 M MRL/lpr high floaters. The 4 M MRL/lpr high floaters were significantly different from all groups.

**Figure 7:**
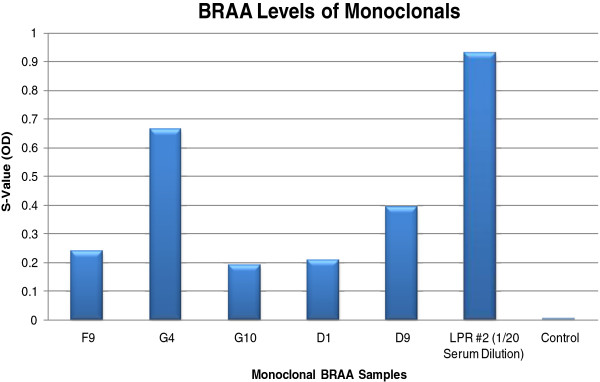
**BRAA ELISA Results of our Potential Pathogenic Monoclonal BRAA.** The graph showed the reactivity of the five potential clones (above 0.1 OD) and MRL/lpr#2. No binding was detected in the control.

Statistical analysis for Figures 1A, 1B, 2, 3, 4, 5 and 6, Additional file 1: Figure S2, Additional file 2: Figure S3 and Additional file 3: Figure S4 were determined using 1-way ANOVA and LSD Post-Hoc analysis (SPSS 16.0 and PASW Statistics 18). Figure 7 was plotted using Microsoft Excel 2007. Microsoft Excel was also used to calculate the mean and standard deviation (SD) for the control C3H/HeJ group and the binding ratios for each of the test groups versus the C3H/HeJ mice. The peptides in the groups were kept for further analysis only if their binding ratios were above the mean plus 0.25 SD, mean plus 1.5 SD or mean plus 2.5 SD depending on the peptide set (the cutoff specific to each peptide set is listed in the Results section).

To determine possible matches between the peptides and potential protein targets, the peptides were aligned individually to all proteins in the mouse proteome, using a gapless local alignment. Alignment scores were summed along each protein and proteins were ranked by their maximum score. The same numbers of peptides were randomly selected from the entire 10 K to estimate the null distribution of these scores. An empirical one-sided p-value was reported based on the percentage of proteins having higher scores from the test peptides vs. the randomly selected peptides. This analysis was performed using an application called Guitope [19]. Halperin and colleagues previously demonstrated that a similar method of aligning random-sequence peptides selected from array experiments has some value in predicting epitopes [28].

## Results

### Goal 1 (Sections 1 and 2): identify possible diagnostic peptides of lupus and CNS-lupus

#### Section 1: study 1 - identify possible diagnostic peptides of lupus and its CNS manifestations

In this section Study 1 was designed to identify possible “diagnostic” antibodies in the sera of 4 month MRL/lpr mice based on the binding intensities of the different peptides compared to the controls (MRL/mp and C3H/HeJ mice). We also identified possible diagnostic peptides of altered behavior in the forced swim test. We called these “diagnostic peptides” since at 4 months the MRL/lpr mice are expected to have lupus-like disease. We used the MRL/mp as a genetic control for the MRL/lpr since they are genetically identical (without the lpr gene) but have late-onset autoimmunity.

#### Possible diagnostic peptides for lupus

##### Immunological assessment and disease activity

We previously showed that increased serum anti-DNA autoantibody levels and spleen weights correspond to disease activity [29,30]. Using 3 randomly selected mice from each group (Figure 1A), ANOVA revealed that anti-DNA autoantibody levels were significantly different between the groups (F = 112.953, p < 0.001). LSD post-hoc analyses at p < 0.001 showed that the MRL/lpr had significantly greater anti-DNA autoantibodies in comparison to both C3H/HeJ and MRL/mp. Regarding spleen weights (Figure 1B), ANOVA showed that there was a significant difference between the groups (F = 18.365, p < 0.003) and post-hoc analysis at p < 0.007 revealed that the MRL/lpr mice had greater spleen weights compared to the controls. The increase in both spleen weights and anti-DNA autoantibody levels indicated that lupus was progressing.

##### Microarray analysis revealing possible diagnostic peptides of lupus

The sera samples from all nine mice were analyzed in triplicates using microarray technology to ensure consistency. Additional file 4: Figure S1 illustrated a representative slide from each group. Additional file 4: Figure S1A and 1B were the no primary control and C3H/HeJ slides, respectively, and little reactivity was detected. Additional file 4: Figure S1C displayed the MRL/lpr serum reactivity. The MRL/mp had lower reactivity than the MRL/lpr (Additional file 4: Figure S1D).

To determine possible diagnostic peptides of lupus, we divided the average binding intensities of the 4 month MRL/lpr by the average binding intensities of the 4 month C3H/HeJ, creating a binding ratio for each peptide. This picked out antibodies favoring autoimmune mice, but to be more conservative in our selection, we selected only those peptides whose ratios were greater than the mean plus 1.5 SD of the binding intensities of the 4 month C3H/HeJ. There were 193 possible diagnostic peptides of lupus.

#### Possible diagnostic peptides of altered behavior in forced swim test

##### Brain-reactive autoantibody levels

BRAA level was used to measure CNS involvement, which has been discussed previously [2,9]. ANOVA (Figure 2) revealed that there was a significant difference between the groups (F = 14, p < 0.005). Post-hoc analysis at p < 0.004 showed that the MRL/lpr had greater levels compared to the controls.

##### Behavioral dysfunction in the forced swim test

In the forced swim test, a significant difference in float time was detected between the groups (Additional file 1: Figure S2) (F = 12.068, p < 0.008). Post-hoc analysis at p < 0.007 revealed increased float times for the MRL/lpr compared to the MRL/mp and C3H/HeJ, indicating CNS involvement (possibly emotional dysfunction) [1,11].

##### Group separation within MRL/LPR for forced swim test

Behavioral heterogeneities have been reported within the MRL/lpr group, i.e., dissimilarity in performance depending on the test [1]. The 4 month MRL/lpr had varying anti-DNA and BRAA levels and diverse binding patterns. Because of these differences, we may be able to identify peptides bound by autoantibodies that may be diagnostic of certain neuropsychiatric manifestations.To test this, we first grouped the mice according to their behavior in the forced swim test and then looked for differences in peptide binding. MRL/lpr #2, #3 and #4 were grouped as high floaters and MRL/lpr #1 and #5 were low floaters (Figure 3). There was an overall significant difference between the groups (F = 9.2, p < 0.008) and post-hoc analysis at p < 0.010 revealed statistically significant differences between the MRL/lpr high floaters compared to the low floaters, MRL/mp and C3H/HeJ. There were no significant differences between the MRL/lpr low floaters and the MRL/mp and C3H/HeJ.

The MRL/lpr were also regrouped based on their anti-DNA autoantibody levels (Additional file 2: Figure S3). The breakdown was similar to the forced swim test (Figure 3). There was a significant difference between the groups (F = 91.176, p < 0.001) and post-hoc analysis at p < 0.004 revealed significant differences between the 4 month MRL/lpr with greater anti-DNA antibody levels compared to the MRL/lpr with lower anti-DNA autoantibody levels, the MRL/mp and C3H/HeJ. Significant differences were detected between the MRL/lpr with lower anti-DNA autoantibody levels and the MRL/mp and C3H/HeJ. This suggests that disease activity (other than BRAA) may be contributing to these behavioral manifestations.

##### Microarray analysis revealing possible diagnostic peptides of altered behavior in the forced swim test

We ran microarray analyses based on the grouping in Figure 3 and selected peptides where the 4 month MRL/lpr high floaters had greater binding intensities than the 4 month MRL/lpr low floaters. Next, the binding intensities of these peptides were divided by their respective 4 month C3H/HeJ binding intensities. We selected only peptides with greater binding intensities than the mean plus 1.5 SD of the binding intensities of the C3H/HeJ. There were 261 possible diagnostic peptides of altered behavior in the forced swim test.

#### Section 2: study 2 - further identify possible diagnostic peptides of lupus and CNS-lupus

In Study 2 we could better suggest which peptides may be diagnostic of lupus and its CNS involvement by comparing the peptide sets generated in Study 1 to those generated in Study 2. Altered behavior in the forced swim test assessed CNS involvement.

#### Identifying possible diagnostic peptides of lupus

##### Disease activity

ANOVA of the quantified anti-DNA autoantibody levels for the 9–10 MRL/lpr and MRL/mp revealed that there was a significant difference between the groups (F = 44.067, p < 0.001). LSD post-hoc analysis at p < 0.001 showed that the 4 month MRL/lpr had significantly greater anti-DNA autoantibody levels compared to the 1.5 and 4 month MRL/mp and 1.5 month MRL/lpr (Figure 4), signifying disease activity [29].

##### Microarray analysis identifying possible diagnostic peptides of lupus

The same type of analysis performed in section 1b of Study 1was repeated here using the 4 month MRL/lpr and the 4 month C3H/HeJ from Study 1. There were 172 possible diagnostic peptides detected in Study 2. When comparing the 193 peptides from Study 1 to the 172 peptides from Study 2, 58 were in common. Since these 58 peptides were reoccurring, this suggests that these 58 peptides may be effective at diagnosing lupus (Table 1).

**Table 1:** Diagnostic peptides of lupus

**Diagnostic peptide sequence of lupus**	**Binding ratios**		**In common with predictive peptides of lupus**	**In common with diagnostic peptides of CNS-lupus**	**In common with predictive peptides of CNS-lupus**
	**Study 1 4MLPR/ 4MC3H**	**Study 2 4 M LPR/ 4MC3H**			
ADGSNWAARHWIPRMPRGSC	3.404555813	2.332923454			
AMSFHRGWDRKYRMSNIGSC	2.599608572	2.484332734			√
AQLGMYGVYRPVEIWPDGSC	2.872747926	2.519910261		√	√
ATDKTRFHFLYDYIRSNGSC	2.399571763	2.880058556			
DDTLYNAHKHLKWFGFIGSC	2.515491313	3.037141329		√	
EATGNDWVITRGGMRRYGSC	2.556966833	3.171502541			
EMNNGRFHRWAQQERHPGSC	2.880990297	2.507493739			
EMSWPRKPWRSKYYHEIGSC	2.353443025	2.435039315	√		√
ENILPTGRDRVAGWYRYGSC	2.825255829	2.675812354	√		
EPKLWFKPRRGGYRHRHGSC	2.679076813	2.434485702		√	
ERIYRDHFIHEHKANIIGSC	2.389911067	2.665777953			√
EWYYDPRGGTGSFYMRTGSC	2.826268307	3.144085944			√
FNRDHREFFEHFGFDEPGSC	4.422628739	2.314403551	√		
FPGDRRSGRAFPEVRWRGSC	2.66924554	2.635674506		√	
FTLMTGKKMIVWDWQRDGSC	2.564037604	2.381362551			√
FWEHHVFHSSRRDGWASGSC	2.951177541	2.464272834			
GLVSRIPSVPKHDEWTFGSC	2.38601487	2.329539073		√	
GRVPQDFNTPSFDRVFWGSC	2.513399666	2.788409089		√	
GWLKAMGPFPWGRLVQNGSC	2.681235945	3.008923213		√	√
IEAMGPSQRYRGRYELIGSC	2.377783579	2.371993113			
IGQRLKGKDENIRFENFGSC	2.347244686	3.594919118		√	√
ILDRRETAWNEHFSKFRGSC	2.781744911	2.979425643		√	
KAMSIHQLANPFDWHFWGSC	2.316559723	2.705671849			
KGYSIRHTEHAWPDIYVGSC	2.579657821	3.104342319			√
KLLMTDFMAKWPRNGWYGSC	3.325866727	2.467180958		√	√
KQHPIYIAHFLGTIVKRGSC	2.850248411	2.447758339			
KVDYVNQWARRRIFMAPGSC	2.933406801	2.826782754		√	
KWLQTQLNSAMYYIRLYGSC	3.442674101	2.816729922			
LAFAWKPDPWQSLVTKFGSC	3.276131888	2.698697705		√	
LFSFKEPQPFMWNKWQQGSC	3.390564552	2.759114763			
LRKISRGIWGMREAGEFGSC	2.476731381	3.603591875			
MFARAHNFDWVKWPLNRGSC	2.705759435	2.921507814			√
MWMSWGWAMLWLNGMMQGSC	4.177408974	2.432118292			
PLVHPWYPTYIPGRHNMGSC	3.630154471	2.495553997			√
PMLFWKWHRQLNQQGRRGSC	2.75721307	2.335852256		√	√
PNPEAWARSFKRWNRKFGSC	3.313709957	3.508821684			√
PSAWEWIPRNQHLNKFRGSC	3.112407959	2.391409291		√	√
PTWRLPPYTDPPKYWHPGSC	4.201093721	3.373945327			
PYRFDWAALPLKKPMWRGSC	2.40624731	2.565057719		√	
QKKPPDYRTWHHPFYNGGSC	2.604969331	2.754487673		√	
QKRWLQLPRNLMWRRETGSC	2.664022568	2.529899901		√	
QRKIFFNYKLHKIWFTAGSC	2.362843141	2.462441712			
QSHWFYDRTKDVYPGRHGSC	3.992759382	2.329563875		√	
RAAMHESLKNWRVYREWGSC	2.388166554	2.725739509		√	√
RPAFDKFADSYWYPPNLGSC	2.471919459	2.483799875			
RRLTKGIIRQYESQLWDGSC	2.38838007	3.049171397			
RTIYRWSQGALSWYMDAGSC	2.443171562	2.802208961			√
SDQVIRGFKDVWQYKWFGSC	3.018394899	2.579167723			
SRDAGLQYPYHRWLTGWGSC	2.452812911	2.732221588			
SRLEQQHFATIPQIWYTGSC	2.462810107	2.571973953			
SRQGLHYNLDGLKPIFPGSC	2.652106822	2.808739776		√	
TLQRTWRRPLLEDLPWWGSC	5.695014524	3.855075257			
VQERMHNRTWKRFGGSMGSC	2.754519694	2.496803929			√
WKPIWHSFHKRRPQILNGSC	2.50380818	2.372369774		√	√
WNGPEWKYSEKSKRILFGSC	2.445136421	2.433467124			√
WSYKYKKKQAWDWPWDPGSC	2.443311505	2.436968425		√	
WTWPSIRFVKGEEYGRFGSC	2.991053751	2.626530982			
YYNVQQVDRWVKLQWGLGSC	2.441114219	2.553038205		√	

#### Identifying possible diagnostic peptides of altered behavior in the forced swim test

##### Brain-reactive autoantibody levels

ANOVA revealed that there was a significant difference in BRAA levels between the groups (F = 9.746, p < 0.001) and LSD post-hoc analysis at p < 0.001 showed that the 4 month MRL/lpr had greater BRAA levels compared to the 1.5 and 4 month MRL/mp and 1.5 month MRL/lpr (Figure 5).

##### Behavior testing

In the forced swim test, there was a significant difference between the groups (F = 11.057, p < 0.001) and post-hoc analysis at p < 0.05 revealed that the 4 month MRL/lpr floated significantly more than the 1.5 and 4 month MRL/mp and 1.5 month MRL/lpr (Additional file 3: Figure S4). This indicated that the 4 month MRL/lpr were displaying altered behavior.

##### Group separation within MRL/lpr based on the forced swim test

Of the nine mice in the 4 month MRL/lpr group, numbers 5, 6, and 7 were grouped as low floaters and the others were classed as high floaters. There was an overall significant difference between the groups (F = 30.253, p < 0.001) and post-hoc analysis at p < 0.001 revealed that the 4 month MRL/lpr high floaters floated significantly more than all the other groups (Figure 6).

##### Microarray analysis validating diagnostic peptides of altered behavior in forced swim test

Similar analysis performed in section 2d of Study 1 was carried out here and 190 possible diagnostic peptides of altered behavior in the forced swim test were detected. A comparison of both peptide sets uncovered 39 in common suggesting that these 39 peptides are likely diagnostic peptides of altered behavior in the forced swim test (Table 2).

**Table 2:** Diagnostic peptides of altered behavior in the forced swim test

**Peptides sequence**	**Binding ratios**		**In common with predictive peptides of lupus**	**In common with diagnostic peptides of lupus**	**In common with predictive peptides of altered behavior in forced swim**
	**Test study 1 4 M LPR high Floaters/ 4MC3H**	**Study 2 4 M LPR high Floaters/ 4MC3H**			
AGAFRERRYKPMMWLHVGSC	2.36	3.24			
AGVRHKFHPYLMQFRRHGSC	2.48	2.44			
AQLGMYGVYRPVEIWPDGSC	3.25	2.58		√	√
DDTLYNAHKHLKWFGFIGSC	2.71	3.21		√	
EKFKRPRWPHLPFTHWDGSC	2.66	2.46			
EPKLWFKPRRGGYRHRHGSC	2.87	3.02		√	
EPSLQVITEYNINFLTIGSC	2.40	3.03			√
EQEDYDDDEEQEQDEDDGSC	2.37	2.36			
ERNRRESDSKERKNYDHGSC	3.25	2.65			
FPGDRRSGRAFPEVRWRGSC	3.27	2.76		√	
GFHGPGMLGKTGRLSYGGSC	2.72	2.49			
GLVSRIPSVPKHDEWTFGSC	2.45	2.49		√	
GRVPQDFNTPSFDRVFWGSC	2.72	2.89		√	
GWLKAMGPFPWGRLVQNGSC	2.93	3.10		√	√
IGQRLKGKDENIRFENFGSC	2.40	4.23		√	√
ILDRRETAWNEHFSKFRGSC	3.37	3.22		√	
IPDGWLKNVYRVRVPWPGSC	2.64	2.34			
IRFVAILVFVIIILIARGSC	2.34	2.95			
KLLMTDFMAKWPRNGWYGSC	3.78	2.72		√	
KTHHSMWKGRITHELFAGSC	2.45	2.52			√
KVDYVNQWARRRIFMAPGSC	3.27	3.35		√	
LAFAWKPDPWQSLVTKFGSC	3.49	2.72			
PMLFWKWHRQLNQQGRRGSC	3.13	2.44		√	
PSAWEWIPRNQHLNKFRGSC	3.38	2.76		√	√
PYRFDWAALPLKKPMWRGSC	2.57	3.16		√	
QKKPPDYRTWHHPFYNGGSC	3.04	3.01		√	
QKRWLQLPRNLMWRRETGSC	2.92	2.77		√	
QRVPIVKWLLWEPRALPGSC	2.99	2.48			
QSAYHNHRMKWRKIGIEGSC	2.47	3.21			
QSHWFYDRTKDVYPGRHGSC	4.79	2.64		√	
RAAMHESLKNWRVYREWGSC	2.49	2.91		√	√
SRQGLHYNLDGLKPIFPGSC	2.77	3.34		√	
SSELDFRKYSFYVHRPDGSC	2.74	2.54			
TLNKRRSWRDGFTADEYGSC	2.31	2.39			
VDARMETFYDMQYPYYLGSC	2.37	2.46			
WKPIWHSFHKRRPQILNGSC	3.05	2.71		√	
WRTKAAMKWQKYQREHRGSC	2.60	2.62			
WSYKYKKKQAWDWPWDPGSC	2.93	2.74		√	
YYNVQQVDRWVKLQWGLGSC	2.67	2.70		√	

### Goal 2 (Sections 3 and 4): identify possible predictive peptides of lupus and CNS-lupus

#### Section 3: identifying possible predictive peptides of lupus

The 4 month MRL/mp from Study 1 and the 1.5 month MRL/lpr from Study 2 were useful in determining possible predictive peptides of lupus since they are lupus-prone but displayed no detectable disease activity (Figures 1A, 1B and 4). Even though the binding intensities were always higher for the 4 month MRL/lpr, the 1.5 month MRL/lpr and 4 month MRL/mp had greater binding intensities for some peptides when compared to the C3H/HeJ control.

We first went back to Study 1 and compared the binding intensities of the 4 month MRL/mp to 4 month C3H/HeJ to identify possible predictive peptides of lupus. Peptides were selected only if their ratios were greater than the mean plus 0.25 SD of the binding intensities of the C3H/HeJ. From this, 143 possible predictive peptides of lupus were identified. In Study 2, we did this same type of analysis using the 1.5 month MRL/lpr but chose peptides where their ratios were greater than the mean plus 1.5 SD of the binding intensities of the C3H/HeJ. In Study 2, 518 possible predictive peptides of lupus were identified. The cut off value was lower for the 4 month MRL/mp in Study 1 because they would develop overt signs of lupus-like disease at a much later time point than the MRL/lpr (develop symptoms shortly after 2 months). When comparing the 143 possible predictive peptides of lupus from Study 1 to the 518 possible predictive peptides from Study 2, 18 peptides were in common and so these 18 peptides were identified as likely predictors of lupus (Table 3).

**Table 3:** Predictive peptides of lupus

**Peptide sequence**	**Binding ratios**		**In common with predictive peptides of altered behavior in forced swim test**	**In common with diagnostic peptides of altered behavior in forced swim test**	**In common with diagnostic peptides of lupus**
	**Study 1 MP/C3H**	**Study 2 1.5 M LPR/C3H**			
DKFHYWMYMLYGINDKIGSC	2.12	2.34			
DKLWKQIWTERHFMSHKGSC	1.57	4.30	√		
DWDSRQINPHIIHHVGRGSC	1.41	2.78			
EEHAHNKLFWWHRSRALGSC	1.61	2.52			
EMSWPRKPWRSKYYHEIGSC	1.46	3.36	√		√
ENILPTGRDRVAGWYRYGSC	1.51	3.10			√
FNRDHREFFEHFGFDEPGSC	1.40	3.61			√
GYNYWIVEWDQDQWLMNGSC	1.39	2.76			
HWKRRHKHKWPKRHPHKGSC	1.92	2.64			
KIWAMRKPRYQYWNQPAGSC	1.41	2.86			√
KWDHGQNGLFPPMHYIPGSC	1.54	3.07			
LEAHYKRSMHAQNWWEAGSC	1.41	2.60			
QYLWWQMLKIEWNSTYAGSC	6.22	2.61			
RHWYQDGSPLLAPVYKVGSC	1.48	3.07			
SYQRENESDEEEKNNEDGSC	1.64	2.38			
VEDNYGVTLRQPKYMGWGSC	1.41	2.36			
WNAMGKWKAMVDKTGDFGSC	2.10	2.40			
WNIHERHRFDQPYDYGHGSC	1.49	2.85			

#### Section 4: identifying possible predictive peptides for altered behavior in the forced swim test

The 1.5 month MRL/lpr from Study 2 were split into two groups identical to the grouping in Figure 6 for the 4 month MRL/lpr and the forced swim test. The logic here is that we know the future performance of these 1.5 month mice at 4 months and so can use them to detect possible predictive peptides of this altered behavior. We averaged the binding intensities for the 1.5 month MRL/lpr in the 2 groups and chose peptides where the binding intensities for the 1.5 month MRL/lpr high floaters were greater than the 1.5 month MRL/lpr low floaters. Then the binding intensities of these peptides were divided by their respective binding from the 4 month C3H/HeJ. We selected only those peptides where their ratios were greater than the mean plus 2.5 SD of the 4 month C3H/HeJ in order to be more conservative in our choice. From this analysis we selected 96 possible predictive peptides of altered behavior in the forced swim test (results not shown due to large number of peptides).

For each of the two-way tests noted above (case vs. control), we performed a leave-one-out cross-validation of the peptide array data using the selected ‘diagnostic’ peptides. We used Support Vector Machines as the classifier (using both SVM in GeneSpring 7.3.1 and in R using the e1071 package). In no case did the cross-validation report >3% error in classification.

### Goal 3 (Sections 5 and 6): identify possible natural antigenic targets

#### Section 5: characterizing potentially pathogenic autoantibodies - identify possible natural protein matches for our peptide sets

Using the sequences from each of the different peptide sets and the Guitope program we suggested possible matches to natural mouse proteins in Table 4.

**Table 4:** Possible natural protein matches for the different peptide sets

**Peptide sets**	**Possible protein matches**
58 diagnostic peptides of lupus	40S ribosomal protein S10 (sp|P63325)
	60S ribosomal protein L22-like 1 (sp|Q9D7S7)
	Histone H3-like centromeric protein A (sp|O35216)
	Follistatin-related protein 4 (sp|Q5STE3)
	H-2 class II histocompatibility antigen, A-D alpha chain (sp|P04228)
39 diagnostic peptides of altered behavior in the forced swim test	Metabotropic glutamate receptor 4 (sp|Q68EF4)
	60s ribosomal protein L22-like 1 (sp|Q9D7S7)
	Calcium/calmodulin-dependent protein kinase kinase 1 (sp|Q8VBY2)
	40S ribosomal protein S9 (sp|Q6ZWN5)
18 possible predictive peptides of lupus	C1q tumor necrosis factor-related protein 6 (sp|Q6IR41)
	Histone H3-like centromeric protein A (sp|O35216)
	Alpha-actinin-2 (sp|Q9JI91)
	60S ribosomal protein L22 (sp|P67984)

#### Section 6: characterizing five created monoclonal brain-reactive autoantibodies

**Figure 8:**
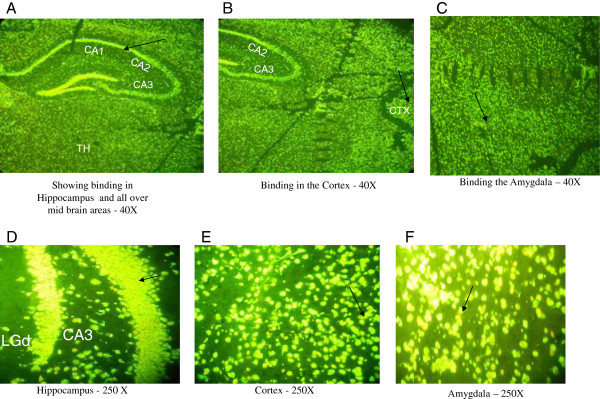
**Immunohistochemical staining using the brain of a control C3H/HeJ mouse and the serum of MRL/lpr #2. (A)** Intense binding detected all over the brain and the hippocampus. **(B)** and **(C)** showed binding in the cortex and amygdala, respectively (40× magnification). **(D)**, **(E)** and **(F)** showed binding in the hippocampus, cortex and amygdala at 250× magnification. Abbreviations: CA, hippocampal regions; LGd, Dorsal part of the lateral geniculate complex; CTX, Cortex.

**Figure 9:**
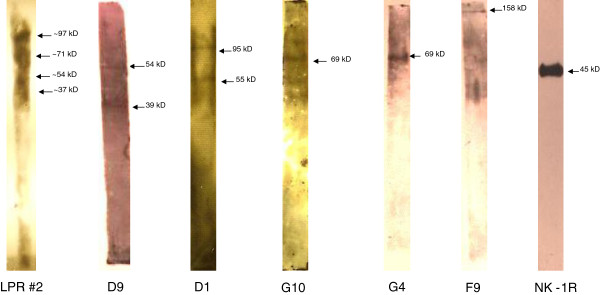
**Western Blotting Results of the Monoclonal BRAA.** Western blotting results showing the banding pattern of the five potential pathogenic monoclonal BRAA, MRL/lpr #2 (mouse used to create the monoclonal BRAA) and the NK-1R positive control. Microsoft Office 2010 Picture Manager was used to adjust the brightness and contrast for some images.

To suggest a possible use of random-sequence peptide microarrays to help identify targets of BRAA, we characterized five potentially “pathogenic” monoclonal BRAA that were created from MRL/lpr #2. These BRAA (labeled F9, G10, G4, D1 and D9) had S-Values above 0.1 OD on the BRAA ELISA (Figure 7). MRL/lpr #2 displayed high float time in the forced swim test (Figure 6), high levels of serum BRAA (Figure 7) and intense fluorescence throughout the brain (Figure 8A), including the cortex (Figure 8B and 8E), amygdala (Figure 8C and 8F) and hippocampus (Figure 8D). In the Western blotting results, which will be discussed below, it should be noted that the bands detected using the serum from MRL/lpr #2 were of similar molecular weight to some of the bands detected using the different monoclonal BRAA created from this mouse (Figure 9).

**Figure 10:**
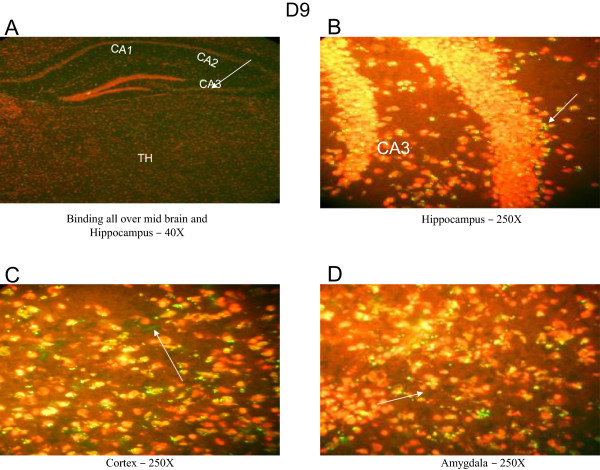
**The brain of a C3H/HeJ mouse was incubated with the Monoclonal BRAA D9. (A)** showed binding all over the brain. **(B)** displayed binding in the hippocampus. **(C)** and **(D)** focused on binding in cortex and amygdala, respectively.

**Figure 11:**
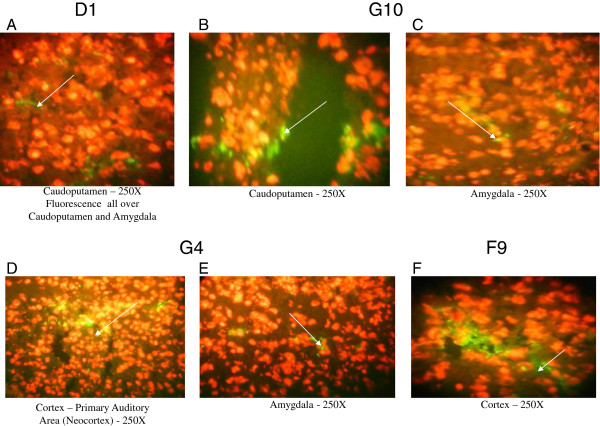
**The above figures showed the binding pattern for the other four monoclonal BRAA D1, G10, G4 and F9. (A)** showed binding in caudoputamen for D1 (There was also binding in amygdala (not shown)). **(B)** and **(C)** showed binding in the caudoputamen and amygdala, respectively for G10. **(D)** and **(E)** showed that for G4 there was binding in the cortex and the amygdala, respectively. **(F)** showed binding in the cortex for F9.

Using our microarray technology (Guitope program and peptide sequences) and Western blotting results, we attempted to determine the mostly likely protein target for the BRAA. In Table 5, we listed six possible protein matches for each of the monoclonals (proteins of interest have been highlighted and some will be discussed). Starting with D9, the approximate molecular weight of its target was 54 kDa and binding was seen all over the brain (Figure 10A), including the hippocampus (Figure 10B), cortex (Figure 10C) and amygdala (Figure 10D). The yellowish/orange color was from propidium iodide staining while the green fluorescence is from the specific antibody. One suggested target was the D (1B) dopamine receptor (DRD5) with a molecular weight of ~54 kDa (The UniProt Consortium, P21918). We also detected a second band at ~39 kDa using the supernatant from D9 (Figure 9). Other researchers have detected a band at ~40 kDa when using anti-D (1B) dopamine receptor antibody, so this receptor may be the protein that we are detecting (Centonze et al., [31]). These two bands could be the same two proteins detected with MRL/lpr #2 at approximately 54 kDa and 37 kDa.Using D1, we detected two bands at 55 kDa and 95 kDa (Figure 9). We observed low levels of binding all over the brain, but greater binding was seen in the caudoputamen and amygdala (Figure 11A). One possible natural protein match could be the gamma-aminobutyric acid receptor subunit rho-1 (GABRR1). GABRR1 has a molecular weight of ~56 kDa (The UniProt Consortium, P24046). The 95 kDa band that was detected could be some kind of modification to the protein or a dimer between this protein and another Gamma-aminobutyric acid receptor subunit. On the antibody data sheet for GABRR1 antibody, two bands were detected at 55 kDa and >90 kDa (ProSci Incorporated, USA). Again, these two bands could be detecting the same two proteins observed in the Western blotting results of MRL/lpr #2 at approximately 97 kDa and 54 kDa.

**Table 5:** Possible natural protein matches for five monoclonal BRAA

**Monoclonal antibody**	**Protein name**
D1	MLX-interacting protein
D1	Endothelin B receptor
D1	Gamma-aminobutyric acid receptor subunit rho-1
D1	CAS1 domain-containing protein 1
D1	Disintegrin and metalloproteinase domain-containing protein 1a
D1	Protein tweety homolog 3
G4	GRB2-associated-binding protein 2
G4	Diacylglycerol kinase epsilon
G4	Synaptotagmin-10
G4	Differentially expressed in FDCP 6
G4	Vesicular glutamate transporter 3
G4	Carbohydrate sulfotransferase 15
D9	Vacuolar protein sorting-associated protein 4B
D9	Transmembrane protein 164
D9	Cytohesin-1
D9	Galanin receptor type 3
D9	D(1B) dopamine receptor
D9	Fas apoptotic inhibitory molecule 2
F9	Serine/threonine-protein kinase TAO2
F9	Epidermal growth factor receptor
F9	Glutamate [NMDA] receptor subunit epsilon-3
F9	Nuclear pore complex protein Nup155
F9	Synaptojanin-2
F9	Astrotactin-1
G10	Autophagy-related protein 9A
G10	Leucine-rich repeat-containing protein 4C
G10	Matrix metalloproteinase-14 Alpha-1,3-mannosyl-glycoprotein 4-beta-N-acetylglucosaminyltransferase A
G10	N-acetylgalactosaminyltransferase 7
G10	Tyrosine-protein kinase Fyn
G10	Highlighted proteins are of interest.

For G10, we detected one band at ~69 kDa (Figure 9) and saw low levels of binding all over the brain, but greater binding was observed in the caudoputamen (Figure 11B) and amygdala (Figure 11C). This band could possibly be the leucine-rich repeat-containing protein 4C. With BRAA G4, a band at ~69 kDa was seen (Figure 9) and we saw binding in cortex (Figure 11D) and the amygdala (Figure 11E) and lower levels of binding all over the brain. This protein could possibly be GRB2-associated-binding protein 2. Even though the targets of G4 and G10 have the same apparent molecular weight, based on the natural protein matches, their identity may be different. With the serum of MRL/lpr #2, a band at ~71 kDa was detected. This could be the same protein detected by either G10 or G4. The only way to be sure is to isolate the proteins and perform mass spectrometry. Lastly, for F9, a band at ~158 kDa was detected (Figure 9) and identified using the microarray as possibly being synaptojanin-2. We saw low levels of binding all over the brain, but greater binding was seen in the cortex for F9 (Figure 11F). No non-specific binding was observed on the secondary only or autofluorescence controls, (Additional file 5: Figure S5A-H).

## Discussion

Our goal was to characterize antibodies in three categories: 1) Diagnostic, 2) Predictive, and 3) Pathogenic. Diagnostic (auto)antibodies may be reliably used for diagnosing a specific disease. Predictive (auto)antibodies can predict the future onset of that disease long before it occurs. Finally, pathogenic (auto)antibodies are responsible for pathogenic mechanisms in the disease causing symptomatology. There is probably a good overlap between these 3 categories, but they can also be different. Thus, diagnostic antibodies need not be predictive (although they can be), and vice versa. Likewise, pathogenic antibodies need not be predictive, although they will almost certainly be diagnostic.

Our first and second goals were to create a detection kit that could diagnose and predict lupus and specific CNS manifestations, using a unique high-throughput microarray technology. This technology has been used in other studies to determine binding patterns specific to that disease, such as Alzheimer’s [16], and we expect that for each disease there will be a different binding pattern that could allow us to distinguish one illness from another ([32,33] and unpublished data). In the future, it would be good to test our microarray technology on other autoimmune diseases to ensure that our tests can distinguish one autoimmune disease from another. Because of the various CNS manifestations and the idea that certain autoantibodies are partly responsible for each manifestation, differences in binding patterns may allow us to distinguish between them in lupus patients.

To begin, we ran the same protocols in two different studies because the first study identified possible diagnostic peptides of lupus and its CNS manifestations and the second study further suggested which peptides may indeed be diagnostic. At the end of Study 2, we identified 58 potential diagnostic peptides of lupus. Of more interest was trying to identify possible diagnostic peptides for specific CNS manifestations. Most MRL/lpr mice will eventually develop lupus, but CNS manifestations differ from one mouse to another [1]. Looking specifically at the forced swim test, we identified 39 possible diagnostic peptides of this altered behavior.

Since we were interested in predicting lupus, in Study 1, we compared the 4 month MRL/mp to the C3H/HeJ since we made the assumption that the MRL/mp would not start developing lupus until 9–12 months of age and therefore at 4 month of age should be pre-symptomatic but having low levels of autoantibodies that are predictive. We assumed that this was also the case for the 1.5 month MRL/lpr used in Study 2 since they would not develop any symptoms until after 2 months of age. Comparison of both studies identified 18 potential predictive peptides of lupus. For the forced swim test, we identified 96 possible predictive peptides of this altered behavior. To further narrow down the true number of predictive peptides, we would need to run another study and administer a comparison of the peptide sets. All these diagnostic and predictive peptide sets only give us indications of which peptides might in fact be diagnostic and predictive, but further studies must be done to support these statements. We would need to run additional test groups to validate our findings. In addition, these studies and techniques give us useful methods and directions to pursue such supportive studies.

When selecting the peptides, we chose only the peptides that were the highest binders; however, it would be interesting in future studies to look at what the lower binding peptides might be telling us. Also, for example, when we selected peptides that may be diagnostic of altered behavior in the forced swim test, we only selected the 39 overlapping peptides between Studies 1 and 2 since diagnostic peptides should reappear from one study to another, however, the non-overlapping peptides were also high binders and therefore are still of interest to us because some of the autoantibodies that are binding these peptides may be diagnostic of another CNS manifestation that we did not investigate.

Due to current inaccurate means of diagnosing lupus and no methods to predict lupus, this microarray technology could help provide proper treatments, improve patient care and add needed therapies. We tested this technology in our mouse model first because this allowed us to not only look at lupus but, of more interest, determine if our technology could distinuguish one CNS manifestation from another. Plus, we wanted to be able to use these techniques in future studies to verify pathogenic BRAA, by injecting the appropriate BRAA into mice (which could not be done in human studies). Another benefit of doing this study in mice first was that we could obtain significant results with fewer samples as in the case of Study 1. A limited set of mice were used in the first study, but a larger number were used in the second study. The purpose of the first study was simply to identify some potential diagnostic peptides, so a large number of mice were not needed. In addition, the statistics were performed on measures that we were very certain would show differences (based on many previous reports in the literature [34,35], including our own [2]) and, in fact, they did. The behavioral test and immunological assessments confirmed that the mice we were using were indeed developing murine lupus as expected. Also, with the mice, we could identify which will develop lupus and CNS manifestations, whereas in human studies this would be difficult and we would need a tremendously larger study to get the same information.

Finally, we have shown in previous studies [36] that human sera can react with murine brain antigen. Thus, we might use murine brain antigens as a diagnostic tool for human CNS-lupus. We found that our microarrays could identify antibody reactivity that demonstrated predictability of specific CNS manifestation in our mouse model. It therefore seems likely that human lupus and its CNS manifestations would also demonstrate a similar trend, although we predict different peptides would be found. Larger trials using human sera samples may determine whether this is true. Expanded human trials would allow us to determine how much personal variability or environmental influences exist in the lupus signature. Mice studies are constrained to clonal animals in identical environments with identical histories. Humans impose much more natural immunological variability. Immunosignaturing has identified both predictive and diagnostic peptides of autoimmune diseases such as diabetes [37], and therefore should be applicable to lupus.

Our third goal was to use the peptide sequences and the Guitope computer analysis program to determine possible natural protein matches, particularly for characterizing the brain antigens which might be mediating CNS manifestations [28]. When looking at the possible diagnostic peptides of lupus, some matches were very interesting since autoantibodies to some of these proteins have been detected in lupus patients [38]. Autoantibodies to the 60S ribosomal protein L12, which is important in protein synthesis, has been detected in 3-28% of lupus patients [38]. Even though this protein is not the 60S ribosomal subunit we detected, it is possible that autoantibodies to our 60S ribosomal protein L22-like 1 can be affecting protein synthesis. Autoantibodies to 40S ribosomal protein S10, which is also important in protein synthesis, have been detected in 11-40% of lupus patients [38]. The histone H3-like centromeric protein A, is like the H3 nucleosome which is important for packaging the DNA in the cell, and autoantibodies to this protein have been detected in 50-90% of lupus patients [38,39]. These anti-histones antibodies are thought to play a role in lupus nephritis, which is one of the manifestations of lupus [38]. The exact mechanism that is occurring and how these autoantibodies are altering body function is not known, but these results help to suggest that these proteins are being affected during disease activity.

When looking at the possible predictive peptides of lupus proteins of interest include C1q tumor necrosis factor-related protein 6 and alpha-actinin-2. The collagen-like region of C1q protein is believed to play a role in lupus nephritis and autoantibodies to this protein occur in about 30-50% of lupus patients [38]. Anti-alpha-actinin-2 antibodies have been detected in patients with lupus nephritis [40]. It was interesting that these researchers detected anti-alpha-actinin-2 antibodies even before lupus nephritis was present. Autoantibodies to histone H3-like centromeric protein A and 60S ribosomal protein L22 were in common with the diagnostic peptides of lupus, so these autoantibodies may be present early on as biomarkers and remain throughout the disease process. The metabotropic glutamate receptor 4 was one of the possible natural protein matches for the potential diagnostic peptides of CNS manifestations (altered behavior in the forced swim test). Since researchers found that using an agonist to this receptor helped to decrease the float time in the forced swim test, this glutamate receptor may play a role as an anti-depressant in CNS-SLE [41].

To further identify potentially affected protein in CNS-SLE we used MRL/lpr #2 from Study 2 to create five monoclonal BRAA. Their possible protein matches are listed in Table 4 and contain some interesting molecules. For example, one possible target for D9 was the D (1B) dopamine receptor, which is expressed in the limbic system and plays a role in neurotransmission; therefore any dysregulation of this receptor would likely result in some neurological deficit, such as affecting memory [42,43]. Other interesting matches include the gamma-aminobutyric acid receptor subunit rho-1, which may play a role in synaptic plasticity in the amygdala (an area of the brain important in emotional dysfunction) [44]; the leucine-rich repeat-containing protein 4C (MAb G10), also known as Netrin-G1 ligand (NGL-1) [45], which may be associated with schizophrenia, and schizophreniform-like behavior is seen in CNS-SLE; the GRB2-associated-binding protein 2 (MAb G4) may be involved in susceptibility to Alzheimer disease [46]; and synaptojanin-2 (MAb F9), which is important in the secretion of vesicles in the synapse and any disruption would affect normal brain functioning [47].

Future experiments, including the use of affinity chromatography where the monoclonal BRAA would be immobilized to a column and then used to isolate its corresponding antigen from mouse brain homogenate, should be employed. One could then identify the antigen using mass spectrometry. These interesting experiments are beyond the scope of the current study, however, we have shown that our microarray technology allows for further characterization of monoclonal BRAA. As a strong test of the autoantibody hypothesis, confirming the role of BRAA in CNS-SLE and the pathogenicity of specific BRAA, it would be necessary to inject these monoclonal BRAA in control mice and see if we can replicate the behavioral dysfunctions.

BRAA are not the only mechanism for triggering CNS involvement in SLE. As seen in Study1, when we split the 4 month MRL/lpr into two groups based on their anti-DNA autoantibody levels, it was noted that this breakdown was the same as for the forced swim test grouping. This suggests factors other than BRAA contribute to the behavioral manifestations in the forced swim test. However, higher anti-DNA autoantibody levels cannot distinguish between several autoimmune diseases so the hope is that the identified diagnostic and prognostic peptides will be more specific to SLE, and more importantly, the different CNS manifestations [48]. Of course, this will need to be verified in future translational studies. A further indication that anti-DNA autoantibody levels is not the best diagnostic marker was seen in our sucrose preference test since the regrouping of the 4 M MRL/lpr into low and high consumers were not the same as the regrouping for the forced swim test or the anti-DNA autoantibody levels (data not included due to inconsistencies across the two studies).

Genetics is a contributing factor to the development of autoimmune diseases such as SLE [49,50]. The major histocompatibility complex in humans (HLA), TNFα, TNFβ, deficiency of complement components and IL10 are just a few examples of genes associated with SLE [49,51]. Alterations in these genes can influence disease onset and progression. The involvement of genetics on the development of lupus-like disease in a mouse model, MBN2, revealed two loci on different chromosomes that suppressed the autoimmune phenotype in male mice [23]. Therefore the influence of such genetic elements on the production of autoantibodies that promoted the different banding patterns discovered using our microarrays need additional investigation. These studies would create further understanding into the mechanisms promoting the pathogenesis of SLE.

One very important asset of using our chip in predicting and diagnosing lupus and its CNS manifestations is its affordability, since this chip is created to be used for any disease. There will be no need to develop a specialized chip just to detect lupus.

## Conclusions

Overall, this random peptide microarray analysis was able to identify both potential predictive and diagnostic markers of lupus and altered behavior in the forced swim test. This technology therefore looks promising as a detection assay for lupus and its neuropsychiatric manifestations. More importantly, as shown above, it could be used to give valuable information about pathogenic mechanisms.

## Abbreviations

CNS: Central nervous system; BRAA: Brain-reactive autoantibodies; SLE: Systemic lupus erythematosus; IP: Intraperitoneal; PBS: Phosphate buffered saline; TBST: Tris-buffered saline with tween; gpr: GenePix results; DRD5: D (1B) dopamine receptor; GABRR1: Gamma-aminobutyric acid receptor subunit rho-1; NGL-1: Netrin-G1 ligand.

## Competing interests

The authors declare that they have no competing interests.

## Authors’ contribution

SW helped design, collect and analyze the data presented in this paper. PS and SH helped design the different experiments and also with the analysis of the data. SW, PS and SH have been involved in the preparation of this manuscript and writing the revisions. All authors approve the final version of this manuscript.

## Supplementary Material

Additional file 1: Figure S2 Study 1- Behavioral Dysfunction (Forced Swim Test). A significant difference in float time was detected (F = 12.068, p < 0.008) and post-hoc analysis at p < 0.007 revealed that the 4 month MRL/lpr had significantly greater float times compared to the MRL/mp and C3H/HeJ. Click here for file

Additional file 2: Figure S3 Study 1 – Group Separation within MRL/lpr by Anti-DNA Autoantibody Levels. The 4 M MRL/lpr mice were also split based on their anti-DNA antibody levels (grouping was similar to Figure 3). There was a significant difference between the groups (F = 91.176, p < 0.001). Utilizing post-hoc analysis at p < 0.004 there was a significant difference between the 4 month MRL/lpr with greater anti-DNA autoantibody levels and the 4 month MRL/lpr with lower anti-DNA autoantibody levels, the MRL/mp and the C3H/HeJ. There was also a significant difference between the MRL/lpr with lower anti-DNA autoantibody levels and the MRL/mp and the C3H/HeJ. Click here for file

Additional file 3: Figure S4 Study 2 - Behavioral Dysfunction (Forced Swim Test). There was an overall significant difference between the groups (F = 11.057, p < 0.001) and post-hoc analysis at p < 0.05 revealed that the 4 M MRL/lpr floated significantly longer than the 1.5 M MRL/lpr, 1.5 M MRL/mp and 4 M MRL/mp. The 1.5 M MRL/mp was significantly different from the 4 M MRL/mp. Click here for file

Additional file 4: Figure S1 Sample peptide binding intensities across pooled samples. This figure demonstrated the intensity pattern across individual mice of different strains. Each green dot is the binding of the serum to an individual peptide. **(A)** Secondary Only Control (only secondary and tertiary antibodies added). **(B)** C3H/HeJ. **(C)** MRL/lpr strain. **(D)** MRL/mp. Click here for file

Additional file 5: Figure S5 Immunohistochemistry Control Slides. The above (orange-yellow) fluorescence is from propidium iodide binding to the cell nuclei. **(A)**, **(B)**, **(C)** and **(D)** showed that there was no binding in most of the brain section, the hippocampus, cortex and amygdala for the secondary only control. **(E)**, **(F)**, **(G)** and **(H)** showed that there was no binding in the whole brain, hippocampus, cortex and amygdala for the auto-fluorescence control. Click here for file
