# Supplementary figures and images for: Diagnosis and early detection of CNS-SLE in MRL/lpr mice using peptide microarrays
Source: BMC Immunol. 2014 Jun 7;15:23. doi: 10.1186/1471-2172-15-23 (PMC4065311; doi:10.1186/1471-2172-15-23)

## Slide 1
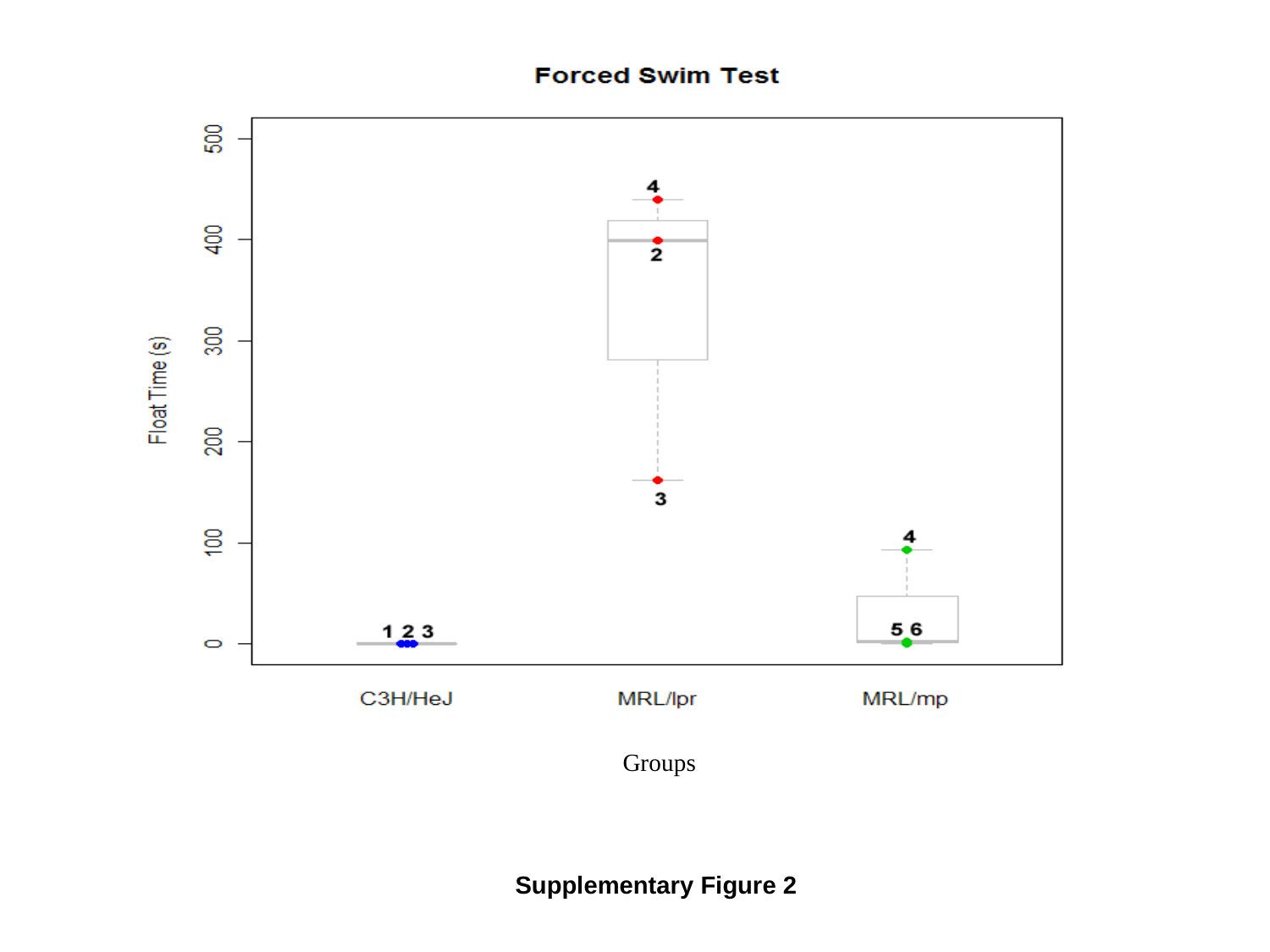

Groups
Supplementary Figure 2

Supplement: Additional file 1: Figure S2 — Study 1- Behavioral Dysfunction (Forced Swim Test). A significant difference in float time was detected (F = 12.068, p < 0.008) and post-hoc analysis at p < 0.007 revealed that the 4 month MRL/lpr had significantly greater float times compared to the MRL/mp and C3H/HeJ. [file 1471-2172-15-23-S1.ppt]

## Slide 1
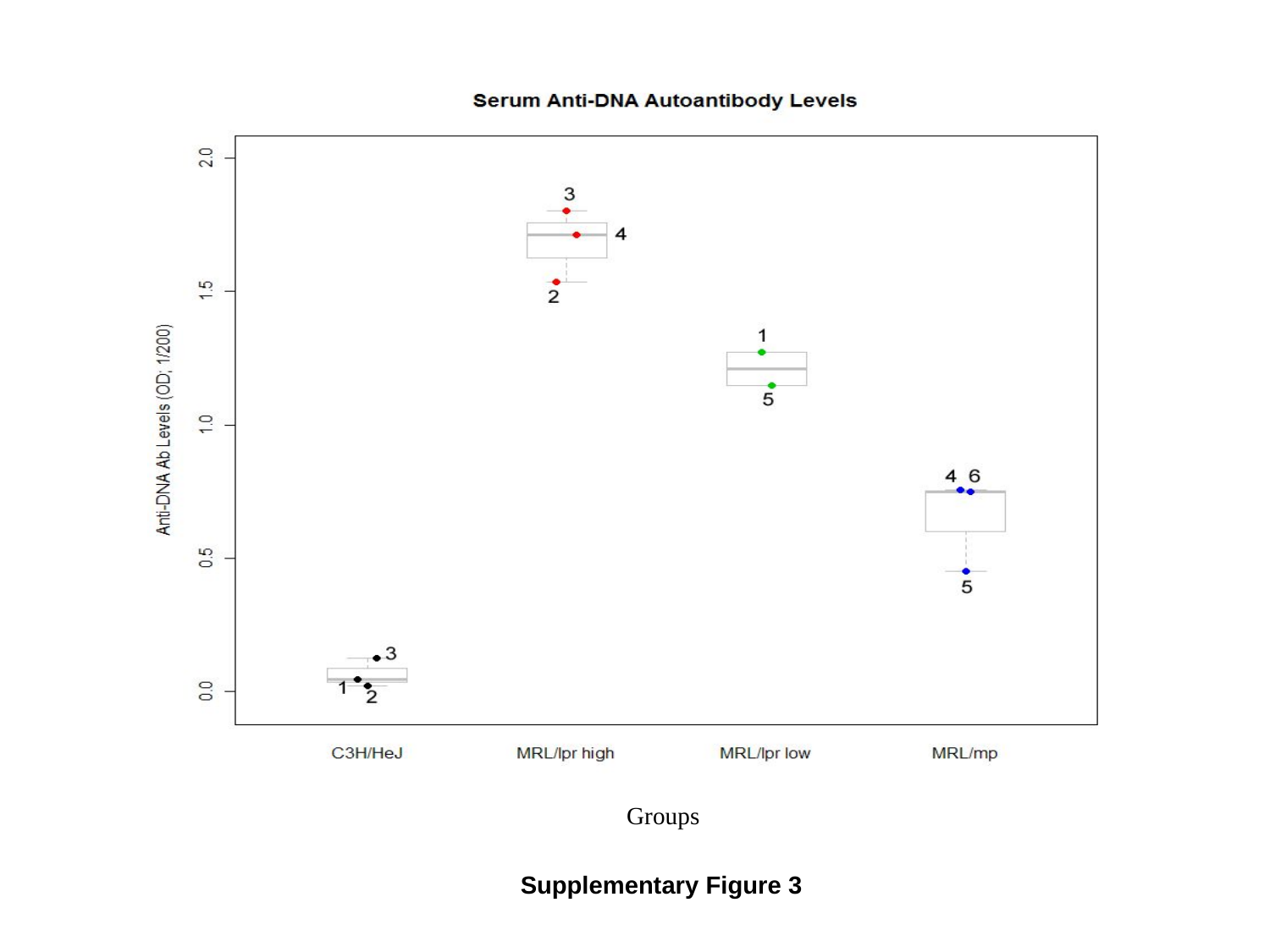

Groups
Supplementary Figure 3

Supplement: Additional file 2: Figure S3 — Study 1 – Group Separation within MRL/lpr by Anti-DNA Autoantibody Levels. The 4 M MRL/lpr mice were also split based on their anti-DNA antibody levels (grouping was similar to Figure 3). There was a significant difference between the groups (F = 91.176, p < 0.001). Utilizing post-hoc analysis at p < 0.004 there was a significant difference between the 4 month MRL/lpr with greater anti-DNA autoantibody levels and the 4 month MRL/lpr with lower anti-DNA autoantibody levels, the MRL/mp and the C3H/HeJ. There was also a significant difference between the MRL/lpr with lower anti-DNA autoantibody levels and the MRL/mp and the C3H/HeJ. [file 1471-2172-15-23-S2.ppt]

## Slide 1
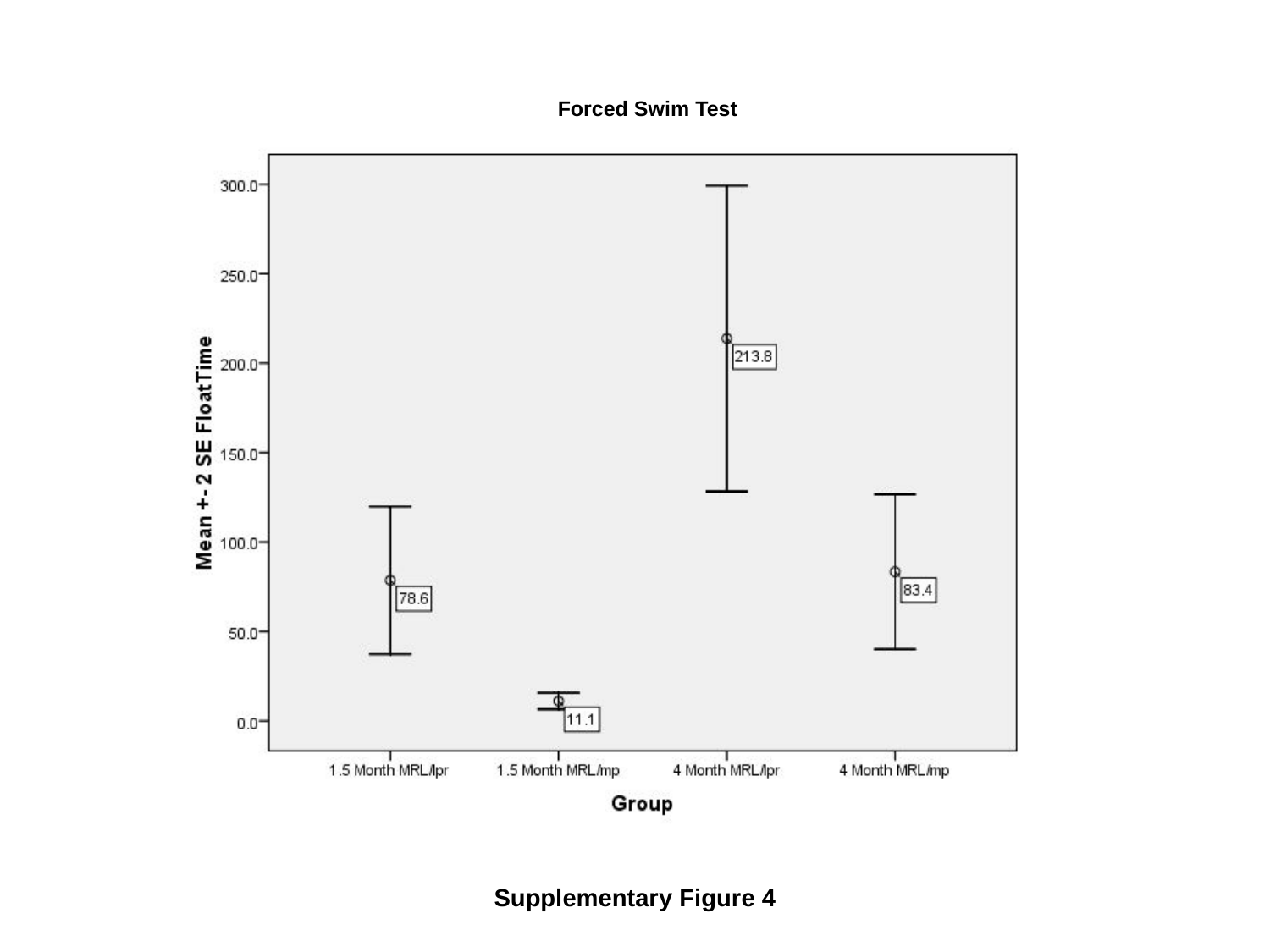

Forced Swim Test
Supplementary Figure 4

Supplement: Additional file 3: Figure S4 — Study 2 - Behavioral Dysfunction (Forced Swim Test). There was an overall significant difference between the groups (F = 11.057, p < 0.001) and post-hoc analysis at p < 0.05 revealed that the 4 M MRL/lpr floated significantly longer than the 1.5 M MRL/lpr, 1.5 M MRL/mp and 4 M MRL/mp. The 1.5 M MRL/mp was significantly different from the 4 M MRL/mp. [file 1471-2172-15-23-S3.ppt]

## Slide 1
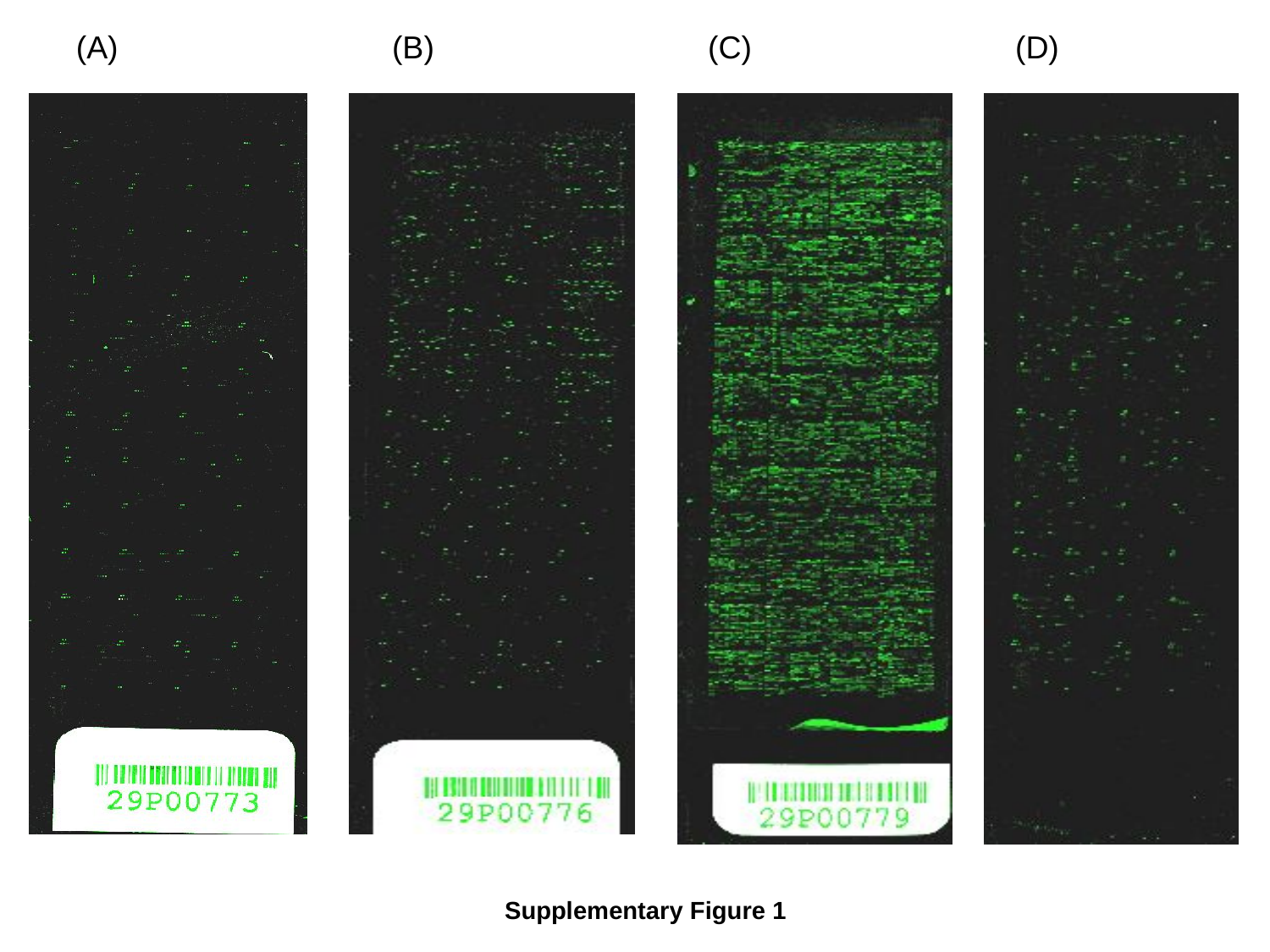

(A)	 	 (B)		 (C) 	 (D)
Supplementary Figure 1

Supplement: Additional file 4: Figure S1 — Sample peptide binding intensities across pooled samples. This figure demonstrated the intensity pattern across individual mice of different strains. Each green dot is the binding of the serum to an individual peptide. (A) Secondary Only Control (only secondary and tertiary antibodies added). (B) C3H/HeJ. (C) MRL/lpr strain. (D) MRL/mp. [file 1471-2172-15-23-S4.ppt]
