# Supplementary material for: Diagnosis and early detection of CNS-SLE in MRL/lpr mice using peptide microarrays
Source: BMC Immunol. 2014 Jun 7;15:23. doi: 10.1186/1471-2172-15-23 (PMC4065311; doi:10.1186/1471-2172-15-23)
Supplement: Additional file 5: Figure S5 — Immunohistochemistry Control Slides. The above (orange-yellow) fluorescence is from propidium iodide binding to the cell nuclei. (A), (B), (C) and (D) showed that there was no binding in most of the brain section, the hippocampus, cortex and amygdala for the secondary only control. (E), (F), (G) and (H) showed that there was no binding in the whole brain, hippocampus, cortex and amygdala for the auto-fluorescence control. [file 1471-2172-15-23-S5.ppt]

## Slide 1
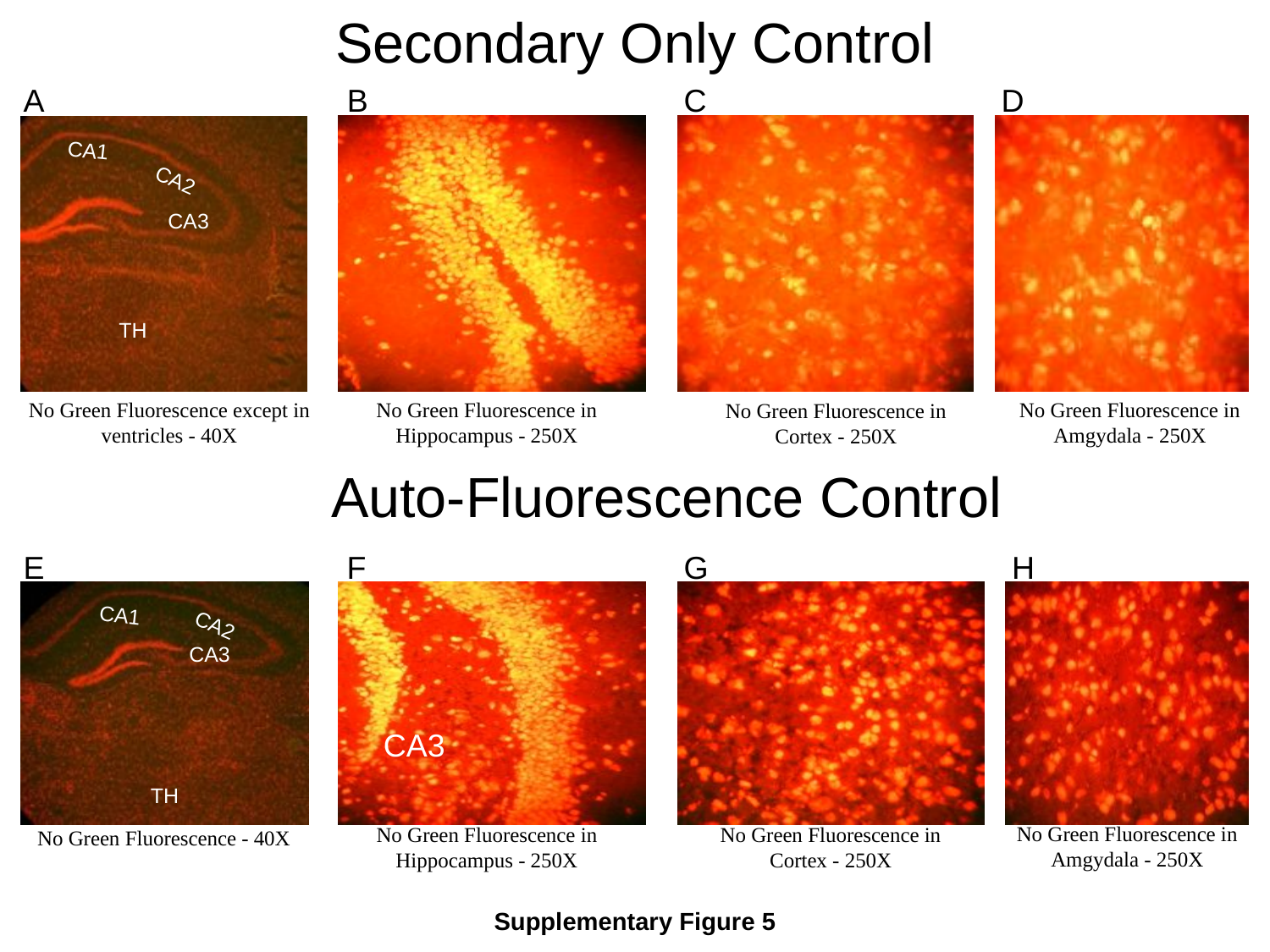

# Secondary Only Control
A
B
C
D
CA1
CA2
CA3
TH
No Green Fluorescence except in ventricles - 40X
No Green Fluorescence in Hippocampus - 250X
No Green Fluorescence in Amgydala - 250X
No Green Fluorescence in Cortex - 250X
Auto-Fluorescence Control
E
F
G
H
CA1
CA2
CA3
CA3
TH
No Green Fluorescence in Amgydala - 250X
No Green Fluorescence in Hippocampus - 250X
No Green Fluorescence in Cortex - 250X
No Green Fluorescence - 40X
Supplementary Figure 5
